# Photodynamic Theranostics of Central Lung Cancer: Capabilities of Early Diagnosis and Minimally Invasive Therapy (Review)

**DOI:** 10.17691/stm2021.13.6.09

**Published:** 2021-12-28

**Authors:** G.V. Papayan, A.L. Akopov

**Affiliations:** Senior Researcher, Laser Medicine Center; Pavlov First Saint Petersburg State Medical University, 6-8 L’va Tolstogo St., Saint Petersburg, 197022, Russia; Senior Researcher, Research Department of Myocardial Microcirculation and Metabolism; Almazov National Medical Research Centre, 2 Akkuratova St., Saint Petersburg, 197341, Russia; Professor, Head of Thoracic Surgery Department, Research Institute for Surgery and Emergency Medicine; Pavlov First Saint Petersburg State Medical University, 6-8 L’va Tolstogo St., Saint Petersburg, 197022, Russia;

**Keywords:** central lung cancer, lung cancer screening, fluorescent diagnosis, photosensitizers, chlorin е6, photodynamic therapy, indocyanine green, photodynamic theranostics

## Abstract

**The aim of the study** was to assess the prospects for central lung cancer (CLC) screening using fluorescent diagnostics and its treatment by endobronchial photodynamic therapy (PDT).

Bronchoscopic fluorescent diagnostics using chlorin e6 photosensitizers and a developed instrumental system enable to reveal tumor changes in large bronchi mucosa at early stages, and a developed PDT technique performed under fluorescent control helps achieve personalized treatment. Such an approach is considered as a theranostic technique — photodynamic theranostics.

central lung cancer screening requires a fluorescent dye characterized by availability and can be used directly within the examination. Indocyanine green can be used as a dye, its peculiarity is the necessity to excite and record fluorescence in the near-infrared (NIR) wavelength band. First experiments using NIR bands to diagnose a bronchoscopic system showed the detectability of tumor areas using on-site bronchoscopic photodynamic theranostics, which consists in NIR imaging of tumor foci when a standard dose of indocyanine green is administered during the examination.

**Conclusion:**

Further progress of early diagnostics and minimally invasive CLC therapy will be determined by the development of new photosensitizers, which should be characterized by a high absorption band in NIR area, quick accumulation in a tumor, high yield of single oxygen in NIR illumination, bright fluorescence, high potential in terms of the induction of an anti-tumor immune response.

## Introduction

Lung cancer is one of the leading death causes from oncology diseases, and 5-year survival of such patients is no more than 10–15% [1]. A key factor of efficiency improvement in oncology patients is early diagnostics [2, 3]. The basic technique of early lung cancer diagnosis is computed tomography, however, even the use of the most up-to-date tomographic scanner cannot solve the problem of detecting latent radiolucent forms of central lung cancer (CLC) amounting 30% of total lung cancer cases [4]. Currently, there are no early CLC diagnostic methods that could be recommended to use in a routine clinical practice, apart from bronchoscopy performed in white light, which has relatively low sensitivity (0.70) and specificity (0.78) [5]. Therefore, such bronchoscopy usually fails to early detect tumor areas in main bronchi/ trachea that can lead to dramatic consequences shown in mortality statistics [6].

One of the prospective techniques of early diagnostics in CLC and premalignant lesions is bronchoscopy in fluorescent light [7]. If bronchus lesion can be early detected by using bronchoscopy, then the tumor can be radically treated using a minimally invasive endoscopic surgery.

The present review is devoted to the assessment of CLC screening prospects using fluorescent diagnostics, and its treatment by endobronchial photodynamic therapy (FDT).

## Central lung cancer diagnostics

Recently, there have been suggested to use different optical techniques combined with routine bronchoscopy to empower diagnostics in early pre-clinical CLC stages; they are narrow-band imaging, high magnification video bronchoscopy, optical coherence tomography, confocal laser microscopy, Raman spectroscopy. Unfortunately, these techniques widely used in detecting malignancies of other sites have not proved their efficiency in early CLC diagnosis yet [8–10]. Moreover, currently, no studies on early CLC diagnosis are being carried out. The techniques based on the analysis of blood samples and inhaled air; which are now at a developmental stage, can appear interesting from the point of view of mass survey [11, 12]. However, since the methods are unable to detect lesion sites, these methods should be followed by certain imaging techniques anyway.

One of promising techniques in this field is bronchoscopy in fluorescent light [6, 13]. In the early 1990s, high hopes were put on autofluorescence imaging: the first findings [14, 15] suggested greater capabilities to detect bronchial mucous lesions suspicious of early cancer compared to the white-light examination. Autofluorescent bronchoscopy attracted scientists, primarily, by its simplicity, since it required no medicines to be administered, and was able to detect a lesion in the negative optical contrast area without a detailed image analysis. It could serve as a basis for commercial availability of certain instrumental systems [7, 16]. Subsequently, however, autofluorescent diagnostics was demonstrated to have rather low specificity (0.67) [5] despite its very high sensitivity (0.92); currently, the method is rarely used [17–19].

Bronchoscopy in induced fluorescence light using exogenetic substances seems to be the more promising technique for early diagnostics of CLC and precancerous lesions. Fluorescent diagnostics based on induced fluorescence is sometimes called drug or photodynamic diagnosis to distinguish it from autofluorescent diagnostics. In respect to CLC, the diagnosis can be applied using second-generation photosensitizers, among these are 5-aminolevulinic acid (5-ALA) and chlorin preparations. 5-ALA is an endogenous protoporphyrin IX synthesis precursor, and its excess in the body results in its accumulation in intensively dividing cells that can be successfully used in neurosurgery to remove glioblastomas under fluorescence control [20]. However, numerous attempts to use 5-ALA induced fluorescence for early CLC diagnosis failed due to low sensitivity and specificity [21–23].

Chlorin e6-based photosensitizers appeared to be appropriate for diagnostic purposes. When excited by ultraviolet and violet rays, they provide high color contrast when imaging various tumors; it enables to easily distinguish them from healthy tissues by a characteristic red fluorescence [24, 25]. In addition, these photosensitizers are widely used in antitumor PDT due to high quantum output of single oxygen and a strong absorption band in a red region [26].

Photodynamic diagnostics based on chlorin fluorescence can be performed using the system meant for autofluorescent diagnosis since excitement wavelengths (400 nm) used in them suit well for all chlorin photosensitizers. The experience of applying such systems, e.g. SAFE-3000 (Pentax, Japan), enabled Japanese researchers achieve high-level imaging of endobronchial mass lesions using a chlorin preparation NPe6 [27–29]. Our experience in working with similar Russian photosensitizers (Fotoditazin or Radachlorin) proves their findings [30].

## Theranostic approach

In recent years, different medical fields practice an “image-guided surgery and therapy” principle. As applied to CLC, the principle can be based on fluorescent imaging and PDT when one chemical agent is administered [13]. The main conditions for PDT success rate are a significant number of photosensitizers in tumor tissue, sufficient oxygen consumption, as well as optimal amount and light energy supply accuracy when performing PDT [31–38].

We have developed and studied a fluorescent technique for PDT efficiency control, it enables to combine diagnostic and treatment components within a procedure, and therapeutic intervention parameters can be corrected on a real-time basis depending on diagnostic information obtained [30, 39]. Such approach suitable to personalization tendency in current medicine can be considered as theranostics (therapy + diagnostics) variant and called photodynamic theranostics. It presupposes the use of two wavelengths of radiation corresponding to absorption maximum of a chlorin photosensitizer in the regions of 398–410 and 660–665 nm [40]. Chlorin e6 red fluorescent imaging in tissues with an increased concentration of the photosensitizer makes it easy to choose a radiation area and its efficiency monitoring after red fluorescent termination due to photobleaching. A photodynamic effect consists of the following: a photosensitizer in the radiation area completely fades, although in most cases, some time later, after discontinuing therapeutic radiation, red fluorescence appears again at a discolored site. Such an effect of “photo-building up” is likely to be related to the accumulation of a new sensitizer portion in tumor tissue due to its extravasation from blood vessels destroyed during PDT; it enables to lengthen a photodynamic effect [30]. If before PDT no fluorescence of the lesion was found, i.e. not enough photosensitizer has been accumulated in tumor tissue, such patient might not need PDT, since the treatment will have no effect [40].

Thus, owing to fluorescent diagnostics, PDT personalization can be achieved depending on tumor biological characteristics in each specific case. And the detectability of chlorin fluorescence is rather high: 43 (96%) from 45 examined patients with stage II–IV central lung non-small cell cancer were found to have bright fluorescence in the tumor stenosis area [30].

### Video endoscopic system for photodynamic theranostics of central lung cancer

To implement theranostic technologies in CLC therapy, we developed a special apparatus system, which is a bronchofiberscope-based multimodal platform with laser light and a digital camera mounted on the endoscope eye lens [41–43]. Laser, 405-nm wavelength, induced visible fluorescence excitement; for photodynamic radiation, we used a 660-nm laser. RGB lasers were applied to make observations in the reflected white light. All diagnostic radiations were delivered to an endoscope lighting canal through the same monofiber. For a photodynamic effect, an additional light guide was introduced in a forceps aperture. For simultaneous imaging of two views (e.g., in reflected white light and fluorescent light), we used a time-shared switch on of light sources synchronically with a camera and the display of obtained images using a special software program [41–43].

**Figure 1.**
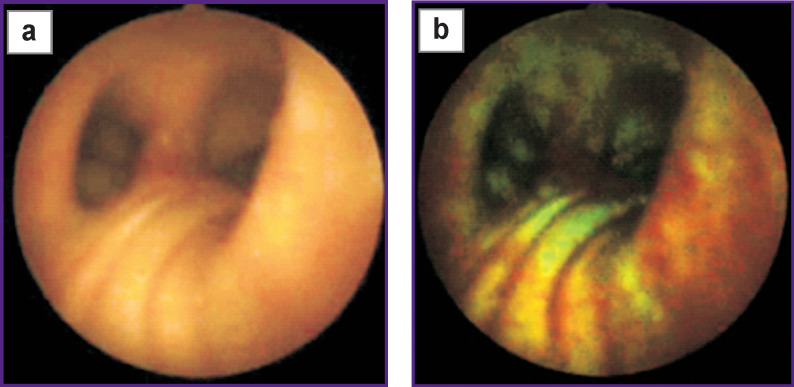
Paired photos of bronchial mucous area in the white light (а) and in fluorescent light (b) in a patient with central squamous cell lung cancer

The presented photos (Figure 1) illustrate chlorin e6 fluorescent visibility: a tumor area invisible in normal (visible) light is clearly seen in fluorescent light due to a red component. In present review, Radachlorin (dose of 1 mg per human body mass kg) was used as a photosensitizer: it was administered intravenously 2 h before the surgery. A pathology report said the material taken from the red fluorescent area by target biopsy was lung squamous cell carcinoma.

Despite high detecting efficiency of chlorin fluorescence, as for diagnostics is concerned, and to be more precise — the diagnostics of preclinical CLC, the use of such photosensitizer is still not reasonable, since it requires pre-administration of the agent that prevents from using the technique as a screening one. For early detection, there needed a fluorescent agent characterized by better availability and the possibility to use it during the examination. Indocyanine green (ICG) can be used for these purposes as it meets the requirements.

### Indocyanine green

Fundamental medical literature of the last years has been discussing the availability of an accumulation mechanism in tumor tissue for diagnostic purposes and target therapy due to the enhanced permeability and retention (EPR) effect, which is related to vascular immaturity resulting from neoangiogenesis [44]. Exogenous molecules are able to permeate through such vessels to tumor tissue and stay there. One of the substances exhibiting such property is ICG. It is being used rather widely in clinical practice to assess liver functions, as well as a contrast agent in ophthalmology. In recent years, the scope of tasks ICG can solve has significantly extended. By means of ICG, lymphography, bile duct imaging, and mapping of sentinel nodes in different malignant tumors being performed; the blood supply quality of transplants, the leak integrity of vascular anastomoses, etc. being determined [45–48]. Characteristic ICG application is the necessity to excite and register fluorescence in near-infrared (NIR) spectrum that requires special equipment available [49].

The main advantages for work in NIR spectrum are as follows: deep penetration of such radiation into human tissue compared to visible light, capability to work in light premises, high contrast due to low autofluorescence. When entering blood flow, ICG quickly binds plasma proteins, 95% of ICG is transported by β-lipoproteins. It is eliminated from blood in two phases [50]. Elimination half-life in the first stage is 3–4 min, in the second — 60–80 min.

Organic nanocarriers for ICG are nature nanoparticles, such as serum albumin (albumin human, AH) [51, 52]. ICG binding to albumin results in the improvement of its characteristic as a fluorescent marker — its fluorescent intensity increases. The study of ICG bound to AH solution showed such binding to be able to result in ICG fluorescence intensity growing by 180 times in relation to its water solution if molecule ratio is AH/ICG ≥1 [53]. Another effective property of ICG + AH is its high stability. In contrast to aqueous ICG, which quickly loses its fluorescent properties due to its tendency for clustering, and requires its preparation immediately before use, ICG  +  AH does not lose fluorescent properties at least within a month, on condition that it is stored at 4°С [53].

The experiments on rats, using ICG-fluorescence, managed to image the subcutaneously grafted tumor areas (Pliss lymphosarcoma) with brightness and contrast, the contrast coefficient being significantly higher 1–3 h after administration when using ICG + AH than when using ICG alone: 3.0–4.9 (ICG) versus 4.6–6.1 (ICG + AH). ICG solution combined with albumin remained in pathological tissues within at least 72 h, and the experiment with double staining (ICG + Radachlorin) found noticeable mismatch of maximum fluorescent areas that can be explained by the difference in selective accumulation mechanisms [53].

Fluorescent ICG imaging based on EPR effect has been used in a variety of clinical studies including lung cancer studies [54–57]. These works used the method developed in the USA and called TumorGlow when ICG is administered systematically, 24 h before the examination, and at a very large dose: 5 mg per human body mass kg; it exceeds a dose usually used in ICG angiography by more than tenfold. And it was followed by the significant rise in the diagnostic procedure price; in addition, it increases the risk of adverse reactions. Therefore, TumorGlow technique is inapplicable for extensive screening assays both on medical grounds and economical reasons.

We succeeded in attaining bronchoscopic imaging of malignant tumor areas in endobronchial masses using the minimal dose of ICG (0.1–0.2 mg/kg) and AH administered intravenously immediately before the surgery or intraoperatively [41]. It is rather complicated to explain the present effect, when it was possible to speed up ICG imaging of bronchial tumors, since in case of other tumor sites both in experiments [53] and under clinical conditions [58] the procedure failed. For ICG fluorescence imaging, the above-described video bronchoscopic system was upgraded by using a laser, 808-nm wavelength, as well as the elements necessary to record NIR fluorescence [39, 59].

**Figure 2.**
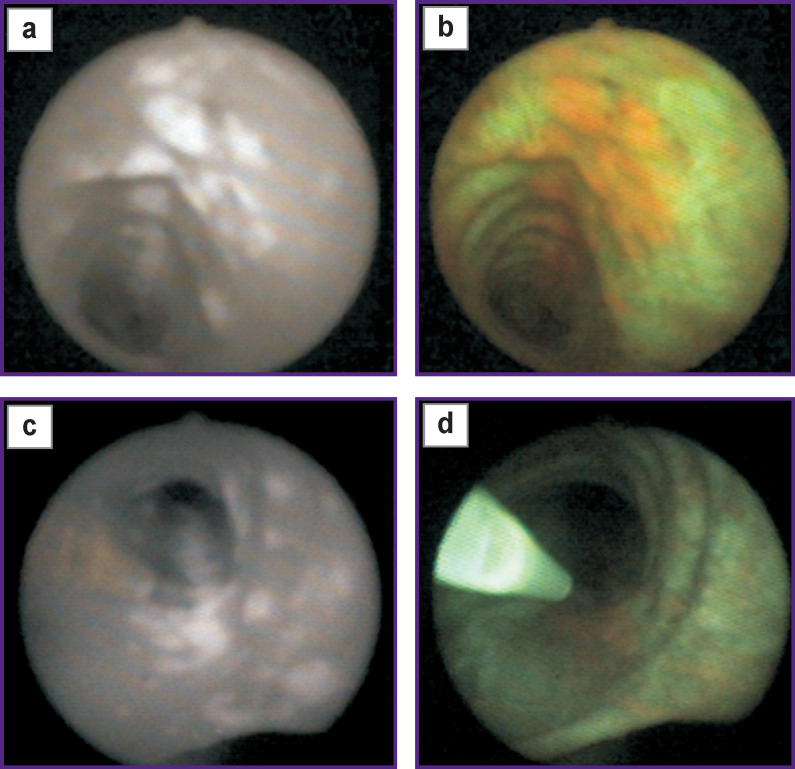
Bronchial mucosa photos taken in double fluorescent staining (indocyanine green + Radachlorin) in near-infrared light (а), (c) and visible (b), (d) fluorescence prior to (а), (b) and at the end of photodynamic radiation (c), (d)

Particular interest is provoked by a series of clinical experiments on administering two fluorescent agents to a patient: Radachlorin (2 h before examination) and ICG (during bronchoscopy, 1 min prior to fluorescent imaging) [39]. Contrast ICG fluorescence (Figure 2 (а)) can image all tumor nodes, which are detected using Radachlorin alone (Figure 2 (b)). Moreover, ICG fluorescence enables us to see the nodes, which are hardly visible in chlorin fluorescent light.

After fluorescent diagnosis stage, which lasted about 5 min, patients underwent PDT using a 660-nm wavelength laser. The photos (Figure 2 (c), (d)) demonstrate the picture at the end of a treatment radiation when a red component of fluorescence became almost invisible resulting from photobleaching, while NIR picture remained unchanged. The feature of using ICG in the present study consists in the fact that NIR imaging was performed at minimal ICG doses, the agent is administered during the examination. The technique was suggested to be called on-site bronchoscopic photodynamic theranostics (OS-BPT) in order to distinguish it from the techniques using larger intervals between agent administration and imaging initiation [39]. Early CLC screening prospects can be based on OS-BPT implementation.

### Photodynamic theranostics in NIR spectrum

Due to the fact that the same agent can be used for diagnosis and treatment, it is reasonable to combine fluorescent diagnostics of early CLC in NIR range and endobronchial minimally invasive treatment within the framework of a single procedure. ICG properties as a photosensitizer both in a solution and as a part of nanoparticles have been studied by many researchers; however, the data on its possible application for PDT are contradictory [60–62]. We also investigated the question in the experiments on a grafted rat tumor (Pliss lymphosarcoma) [53]. An attempt to cure 21 animals using 808 nm-wavelength laser (a light dose of 450–850 J/cm^2^) resulted in tumor growth inhibition in 12 rats (57%) and complete tumor elimination in 2 rats (10%). The findings suggest ICG be a moderate photosensitizer and it can be used both for photodynamic diagnosis and PDT. However, by efficiency, it is inferior to chlorin e6 that is likely to be explained by low quantum efficiency of single oxygen (0.12–0.21 — in ICG [61] and 0.77 — in chlorin e6) [26]. Therefore, the use of ICG as a photosensitizer for PDT of central lung cancer is unlikely to be efficient.

The situation can change if new NIR substances with higher single oxygen yield and higher capability for molecular targeting appear. To enhance the diagnostic specificity of primary non-small cells lung cancer, Predina et al. [57] used OTL38 — a fluorescent-contrast agent, which is conjugated with NIR staining S0456 folate, selectively binding to folic acid receptor α. The receptor is slightly marked or can be absent in healthy tissues, although it is found in some cancer forms including 86% lung adenocarcinomas. For that purpose, conjugated NIR photosensitizers with monoclonal antibodies can be used instead of receptor ligands [63–65].

### PDT-mediated anticancer immune response

One more promising technique in oncology is related to possible antitumor immune response stimulation by PDT. The interest in cancer immunotherapy has significantly increased due to the development of immune checkpoint inhibitors, which enable to achieve a breakthrough in the treatment of a wide range of malignancies including lung cancer. The researches on immune responses after PDT carried out over the last years showed that a photodynamic effect can be successfully used both for tumor elimination and body immune system reinforcement.

Currently, over a hundred and a half scientific articles and a considerable number of special reviews have been devoted to PDT-mediated immunotherapy, or immune PDT (photodynamic immunotherapy) [66–77]. Unfortunately, just a few studies are clinical [78–86], while the overwhelming majority of them were carried out in the form of experiments including those with lung cancer modeling [87–91]. The findings suggest a local PDT effect on a tumor to result in the induction of a systemic antitumor immune response, which enables to control tumor growth beyond a treatment area, and, therefore, it has the potential for metastases treatment. Usuda et al. [92] comparing PDT antitumor effects with various photosensitizers for CLC therapy, demonstrated that due to systemic immune response induction, a chlorin photosensitizer had more profound antitumor effect than Photofrin, and complete response frequency after PDT with NPe6 was significantly higher than after PDT with Photofrin. Furthermore, PDT’s immune effect on tumor can fail. The best results are provided by two-stage therapy. At the first stage, low-density energy radiation is used, which due to the so-called vascular PDT results in tumor-specific neoantigen release stimulating adaptive immunity; and at the second stage, which is carried out several hours or days later depending on photosensitizer type, a primary tumor is destroyed by high-density energy radiation by standard cellular PDT [93]. An immune response in PDT was demonstrated to be received even at a single exposure using a new photosensitizer Redaporfin (synthetical bacteriochlorin developed in Portugal and to be used in biliary tract cancer, which is currently under clinical trials) [94]. It is hoped that such photosensitizers, as well as nanoparticle-based photosensitizers, will be capable of higher T-cells activation in tumor microenvironment — either independently [95], or in a combination in sequential usage of PDT with photothermotherapy [96, 97], or in a combination with immune checkpoint inhibitors [98–101].

## Conclusion

The first findings of photodynamic theranostics are encouraging and enable to count on its potential unlock in relation to early diagnostics and minimally invasive endoscopic treatment of central lung cancer. As for now, the second bronchoscopy is required in trachea or large bronchus mucous tumors confirmed by histology (a target bioptate taken during a screening bronchoscopy with indocyanine green). The second bronchoscopy is aimed at treating the previously revealed early lesions using photodynamic therapy with chlorin photosensitizer. A topical issue is that of developing new photosensitizers, which should have a strong absorption band in NIR area, quick accumulation in a tumor, high yield of single oxygen in NIR irradiation, bright fluorescence, and high antitumor immune focus.

If photosensitizers become available in clinical practice, it will enable to significantly improve the capabilities of diagnostics and minimally invasive treatment of central lung cancer using OS-BPT.
